# Circulating 25-Hydroxy-Vitamin D Levels in Menopausal and Postmenopausal Women in Italy: A Comparison of Four Analytical Methods

**DOI:** 10.3390/diseases14070245

**Published:** 2026-07-06

**Authors:** Flaminia Tomassetti, Martina Pelagalli, Federico Cortese, Alfredo Giovannelli, Enrico Maria Carloni, Maria Morello, Eleonora Nicolai, Alessandro Terrinoni, Massimo Pieri, Sergio Bernardini

**Affiliations:** 1 Department of Experimental Medicine, University of Rome Tor Vergata, via Montpellier, 1, 00133 Rome, Italy; flaminia.tomassetti@students.uniroma2.eu (F.T.); alfredo.giovannelli@gmail.com (A.G.); enricomaria.carloni@students.uniroma2.eu (E.M.C.); morello@uniroma2.it (M.M.); alessandro.terrinoni@uniroma2.it (A.T.); bernards@uniroma2.it (S.B.); 2 Department of Biomedicine and Prevention, University of Rome Tor Vergata, via Montpellier, 1, 00133 Rome, Italy; 3 Department of Laboratory Medicine, Tor Vergata University Hospital, viale Oxford, 81, 00133 Rome, Italy; pelagallimartina90@gmail.com; 4 Department of Electronic Engineering, University of Rome Tor Vergata, via del Politecnico, 1, 00133 Rome, Italy; federico.cortese@alumni.uniroma2.eu; 5 Department of Industrial Engineering, University of Rome Tor Vergata, via del Politecnico, 1, 00133 Rome, Italy; 6 Departmental Faculty of Medicine, UniCamillus-Saint Camillus International University of Health and Medical Sciences, via di Sant’Alessandro, 8, 00131 Rome, Italy; eleonora.nicolai@unicamillus.org

**Keywords:** vitamin D, menopause, chemiluminescence assay, LC-MS/MS

## Abstract

Background: Vitamin D is a key regulator of skeletal homeostasis, and hypovitaminosis D is highly prevalent among postmenopausal women, who are at increased risk of osteoporosis, sarcopenia, and related complications. Accurate assessment of serum 25-hydroxyvitamin D [25(OH)D] is therefore essential. However, substantial variability exists among analytical methods, particularly between automated chemiluminescent immunoassays (CLIA) and liquid chromatography–tandem mass spectrometry (LC-MS/MS), the latter considered the reference technique. This study aimed to compare four analytical methods, three CLIA platforms, and LC-MS/MS for measuring circulating 25(OH)D levels in a cohort of menopausal and postmenopausal women. Methods: A total of 425 serum samples from menopausal and postmenopausal women representing the real-world distribution of vitamin D levels in this population were analyzed using three automated CLIA systems and LC-MS/MS. Method comparison, agreement, precision through quality control assessment, total error, and sigma were evaluated. Results: The evaluated CLIA platforms (Abbott, Snibe, and Siemens) showed strong correlation with LC-MS/MS, with r = 0.919, r = 0.978, and r = 0.879. Furthermore, all assays showed excellent precision (CV < 5%), with good-to-acceptable total error (TE) and Sigma-metric performance. Conclusions: In conclusion, these findings demonstrate that while CLIA platforms offer a reliable and precise alternative for routine clinical use, these findings underscore the importance of method selection and result interpretation in the clinical assessment of vitamin D status in postmenopausal women. Furthermore, it highlights the ongoing need to minimize inter-assay variability and ensure consistent vitamin D assessment.

## 1. Introduction

Postmenopausal women frequently exhibit osteopenia or osteoporosis due to aging and estrogen decline, conditions that markedly increase the risk of falls and fractures. Several studies have demonstrated a close association between muscle mass and bone mineral density in this population. Sarcopenia arises from multiple factors, including inadequate nutrition, physical inactivity, reduced protein synthesis, chronic inflammation, and hormonal or cytokine dysregulation [1,2]. Risk factors for low serum Vitamin 25(OH)D include obesity, malabsorption syndromes, certain medications (e.g., anticonvulsants, antiretrovirals), skin aging, limited sun exposure, and institutionalization. Indeed, Vitamin D plays a central role in maintaining a healthy mineralized skeleton and in preventing rickets and osteomalacia [3,4,5]. Serum 25(OH)D, comprising both 25(OH)D_2_ and 25(OH)D_3_, is the established biomarker for assessing vitamin D status and reflects the body’s vitamin D reserve [6]. Severe deficiency is defined by the threshold required to prevent nutritional rickets or osteomalacia, with broad consensus indicating that 400 IU/day and serum 25(OH)D concentrations above 30 nmol/L (12 ng/mL) are sufficient for prevention.

Menopause further increases susceptibility to vitamin D deficiency due to changes in body composition, aging, reduced sun exposure, inadequate dietary intake, and increased adiposity. Evidence increasingly links vitamin D deficiency to menopausal health conditions, particularly postmenopausal osteoporosis [7], and may exacerbate its risks and also the risk of sarcopenia, cardiovascular disease, diabetes, cancer, infections, and neurodegenerative disorders [8,9].

Accurate assessment of serum 25(OH)D is therefore essential in postmenopausal women, who are at elevated risk of skeletal and muscular complications. However, substantial variability exists among analytical methods, particularly between automated chemiluminescent immunoassays (CLIA) and liquid chromatography–tandem mass spectrometry (LC-MS/MS), the reference technique. LC-MS/MS offers high analytical specificity and the ability to distinguish vitamin D_2_ and D_3_ metabolites, but its use is limited by the need for specialized instrumentation, high operational costs, labor-intensive workflows, and the requirement for highly trained personnel. It is not fully automated and has relatively low throughput, making it unsuitable for many routine laboratories. In contrast, CLIA methods provide rapid turnaround times (TAT), high throughput, and full automation, making them more practical for routine diagnostics despite known issues such as cross-reactivity, matrix effects, and potential bias relative to LC-MS/MS.

Given the clinical relevance of vitamin D deficiency in postmenopausal women, evaluating the agreement, bias, and interchangeability of commonly used assays is essential for ensuring reliable diagnosis and appropriate patient management. The aim of this study is therefore to compare four analytical methods, three CLIA platforms Siemens, Abbott, and Snibe assays, and LC-MS/MS for the measurement of circulating 25(OH)D levels in a cohort of postmenopausal women. The study cohort reflects the real-world distribution of vitamin D levels in this population, enhancing the generalizability of the analytical comparison and ensuring the performance of the assays.

## 2. Materials and Methods

### 2.1. Study Population

A total of 425 serum samples from menopausal and postmenopausal women were selected for this study. The samples were collected as residual serum material from routine laboratory activity at the Department of Tor Vergata University Hospital from February to March 2025. Menopausal and postmenopausal status was defined based on age and biochemical evidence of ovarian failure, including low serum estradiol concentrations (typically <20 pg/mL), age, and the date of the last menstruation, according to previously established reference ranges [10,11]. Calcium and parathormone data were collected from the Laboratory Information System, LIS (Modulab, Werfen, Barcelona, Spain). The study protocol was approved by the appropriate Institutional Ethics Committee (protocol number 141/20) and conducted in accordance with the principles of the Declaration of Helsinki. Informed consent was obtained from each participant. The cohort consisted of females, with a mean age of 64 years (range: 46–94 years).

### 2.2. Chemiluminescent Magnetic Immunoassay

The Atellica 25-hydroxyvitamin D [25(OH)D] assay (Siemens HealthCare, Erlangen, Germany) is a competitive Chemiluminescent Microparticle Immunoassay (CMIA). The solid phase of this system consists of paramagnetic particles (PMPs) covalently linked to an anti-fluorescein mouse monoclonal antibody. The immunochemical reaction relies on the competition between endogenous vitamin D and a fluorescein-labeled vitamin D analog for binding sites on an anti25(OH)D mouse monoclonal antibody labeled with acridinium ester (AE). Due to the competitive nature of the assay, there is an inverse relationship between the concentration of vitamin D in the patient sample and the relative light units (RLUs) measured by the analytical platform. Serum samples from postmenopausal women were collected following standard laboratory procedures. Frozen samples were fully thawed, gently mixed, and homogenized before analysis. The assay was performed on the Atellica instrument.

### 2.3. Chemiluminescent Sandwich Immunoassay

The measurement of serum total 25-hydroxyvitamin D, 25(OH)D, including both 25(OH)D_2_ and 25(OH)D_3_, was performed using MAGLUMI 25(OH)D assay developed by Shenzhen New Industries Biomedical Engineering Co., Ltd. (SNIBE, Shenzhen, China). The assay is a two-step sandwich chemiluminescence immunoassay (CLIA) performed on the fully automated MAGLUMI X3 platform (SNIBE, Shenzhen, China). In this assay, the sample was first mixed with buffer containing a displacing reagent to release 25(OH)D from vitamin D binding protein. The released 25(OH)D was then incubated with magnetic microbeads coated with mouse monoclonal anti-25(OH)D antibodies at 37 °C. During this incubation, 25(OH)D specifically bound to the immobilized antibodies, forming primary antigen–antibody immune complexes on the surface of the magnetic microbeads. After magnetic separation, unbound components were removed by a washing cycle. Subsequently, a second anti-25(OH)D antibody labelled with ABEI [N-(4-aminobutyl)-Nethylisoluminol] was added to the reaction mixture. This labelled antibody selectively recognized the 25(OH)D-antibody immune complex formed in the first step, allowing the formation of a bead-bound sandwich complex. After incubation and following magnetic sedimentation, the supernatant was discarded, and another washing cycle was performed to remove unbound labelled antibody. Finally, starter 1 + 2 are added to initiate the chemiluminescent reaction. The emitted light is measured by a photomultiplier and expressed as relative light units (RLU), which are proportional to the concentration of 25-OH vitamin D present in the sample. The principle of the methodology is schematized in Supplementary Material Figure S1. Quantitative 25(OH)D concentrations were calculated using the calibration curve generated according to the manufacturer’s instructions. Calibration was performed using the two-point master curve provided with the kit. All reagents were used ready-to-use. Internal quality control was performed at two concentration levels at the beginning of each analytical session, and only runs with control values within the specified acceptable ranges were included in the analysis.

### 2.4. Chemiluminescent Immunoassay

Total 25-hydroxyvitamin D [25-OH vitamin D] was measured using the Abbott Alinity i^®^ 25-OH Vitamin D assay (Abbott, Chicago, IL, USA), a delayed one-step chemiluminescent microparticle immunoassay (CMIA) designed for quantitative determination in human serum and plasma. In this method, the sample is incubated with anti-vitamin D–coated paramagnetic microparticles and assay diluent, allowing displacement of 25-OH vitamin D from its binding protein and subsequent binding to the solid phase. A vitamin D acridinium-labeled conjugate is then added to form the reaction mixture, followed by a second incubation. After a wash cycle, Pre-Trigger and Trigger solutions are added to initiate the chemiluminescent reaction. The emitted light is measured as relative light units (RLUs), which are directly proportional to the concentration of 25-OH vitamin D present in the sample. Internal quality control materials at three different concentration levels were analyzed at the beginning of each analytical session to ensure assay performance.

### 2.5. Liquid Chromatography–Tandem Mass Spectrometry (LC/MS-MS)

25-hydroxyvitamin D [comprising both 25(OH)D_2_ and 25(OH)D_3_ fractions] was measured in serum specimens via liquid chromatography–tandem mass spectrometry (LC-MS/MS), as reference method. Chromatographic separation and mass detection were performed using the commercial MassChrom^®^ 25(OH)D3/D2 kit (Order No. 62000/1000; Chromsystems Instruments & Chemicals GmbH, Munich, Germany). This integrated system supplied all necessary components for the analytical workflow, including the analytical column, trap column, and the lyophilized samples for calibration curves and controls. Samples were prepared via protein precipitation and the addition of a deuterated isotopically labeled internal standard. Following preparation, samples were subjected to LC-MS/MS analysis using a Shimadzu Nexera X3 (Shimadzu, Kyoto, Japan) high-performance liquid chromatography (HPLC) system coupled to the Sciex Triple Quad^®^ 6500 + MS/MS system (Sciex, Framingham, MA, USA). Chromatographic separation utilized a trap column and an analytical column operated with a binary gradient. All analyses were carried out in positive mode using atmospheric pressure chemical ionization (APCI). For quantitative analyses, multiple reaction monitoring (MRM) was applied. Of the two options available for chromatographic analysis, a binary gradient was used.

Calibration was performed using the lyophilized human serum-based Multilevel Serum Calibrator Set, applying a linear regression model with 1/x weighting. The calibrators present metrological traceability to the standard reference material NIST SRM 972a. Quality control was monitored by including MassCheck^®^ Serum Controls (Chromsystems Instruments & Chemicals GmbH, Munich, Germany) at multiple concentration levels within each analytical run.

The lower limit of quantitation (LLOQ) is 1.0 µg/L for both 25(OH)D_3_ and 25(OH)D_2_. Based on system specifications, intra-assay imprecision (coefficient of variation, CV) is 2.6–3.0% for 25(OH)D_3_ and 2.4–4.9% for 25(OH)D_2_. Inter-assay CV is 5.1–5.4% for 25(OH)D_3_ and 4.3–5.5% for 25(OH)D_2_. Trueness of the measurement was established via equivalence to the Centers for Disease Control and Prevention reference measurement procedure, exhibiting relative recoveries in serum of 95.8–97.1% for 25(OH)D_3_ and 101.8–103.4% for 25(OH)D_2_. Regarding interfering metabolites, this assay does not chromatographically separate 3-epi-25(OH)D_3_ and 3-epi-25(OH)D_2_ from 25(OH)D_3_ and 25(OH)D_2_, respectively. However, in the adult population, the absolute quantity of the C-3 epimer is small, with its fraction generally averaging around 4% to 5% of the total 25(OH)D concentration. Consequently, its co-quantification does not have a clinically significant impact on the routine assessment of vitamin D status in adults [12,13,14].

### 2.6. Comparison Analysis

Passing–Bablok regression analysis was used to compare the three analytical methods, utilizing the nonparametric measure of rank correlation to assess the statistical dependence between the ordered values of two variables and calculating the Spearman rank correlation coefficient (r). In addition, Bland–Altman analysis was conducted as a complementary approach to evaluate the agreement between each assay and the reference method (LC–MS/MS), and the agreement between each CLIA assay, and to examine any potential association between the measurement discrepancies and the actual (true) concentration values.

### 2.7. Precision Study

Precision was assessed in accordance with the CLSI EP15-A3 guideline [15] using the standardized 3 × 5 experimental protocol. Different concentration levels of internal quality control (QCs) materials were analyzed over five consecutive days, with three replicate measurements performed for each concentration level on each day. This protocol evaluates the estimation of within-run and between-day imprecision under routine laboratory operating conditions [16].

### 2.8. Analytical Assay Performance

To evaluate the analytical performance of the CLIA assay, precision data from internal quality controls (QC) and External Quality Assessment (EQA) of the RIQAS program (Randox, UK) of three different platforms, Abbott, Siemens, and Snibe, were used. A one-year longitudinal EQA (n.12 samples) dataset was evaluated. The bias evaluation was defined as:

$$Bias\%=\frac{Laboratory\;result-Target\;value}{Target\;value}\times 100$$

The methodology was based on the application of the formula for calculating Total Error (TE):

$$TE=\left|Bias\%\right|+1.65\;\times \%CV$$

and the formula for calculating the Sigma metric (σ):

$$\sigma =\frac{TEa-\left|Bias\%\right|}{\%CV}$$

where TEa is the Total Allowable Error, set by the Vitamin D Standardization Program (VDSP) as international references: 21.5% [17,18].

### 2.9. Statistical Analysis

To determine the appropriate statistical approach, data distribution was initially evaluated for normality using the Shapiro–Wilk test. Descriptive metrics were then applied to summarize the study population characteristics. Normally distributed data were expressed as mean ± standard deviation (SD), while non-normally distributed data were presented as median and interquartile range (IQR).

To assess the degree of inter-rater agreement between the immunoassays and the reference LC-MS/MS method for the categorization of Vitamin D status, Cohen’s weighted kappa coefficients (κ) with linear weights were calculated. Kappa values were interpreted according to Altman criteria [19]: <0.20 as poor, 0.21–0.40 as fair, 0.41–0.60 as moderate, 0.61–0.80 as good, and 0.81–1.00 as very good agreement.

All data were examined using MedCalc Ver.18.2.18 (MedCalc Software Ltd., Ostend, Belgium).

The statistical significance level established for all tests performed was *p* < 0.05.

## 3. Results

### 3.1. Population

Descriptive analysis of the study cohort revealed a high prevalence of vitamin D deficiency and insufficiency. The majority of participants, 49.6% (211 out of 425), were classified as having a moderate deficiency, followed by severe deficiency in 19.6% of individuals (83 out of 425) and insufficiency in 18.8% of cases (80 out of 425). Collectively, the sample exhibited serum levels below the clinical reference range. Conversely, only 12% (50 out of 425) of the subjects maintained a normal vitamin D status, while cases of toxicity were rare, accounting for only 1 case of the total population. Table 1 illustrates the correlation between serum 25(OH)D levels, calcium, and parathyroid hormone (PTH) concentrations, showing a progressive increase in PTH as vitamin D status declines from normal to severely deficient levels, while calcium remains relatively stable.

### 3.2. Method Comparison

**Figure 1:**
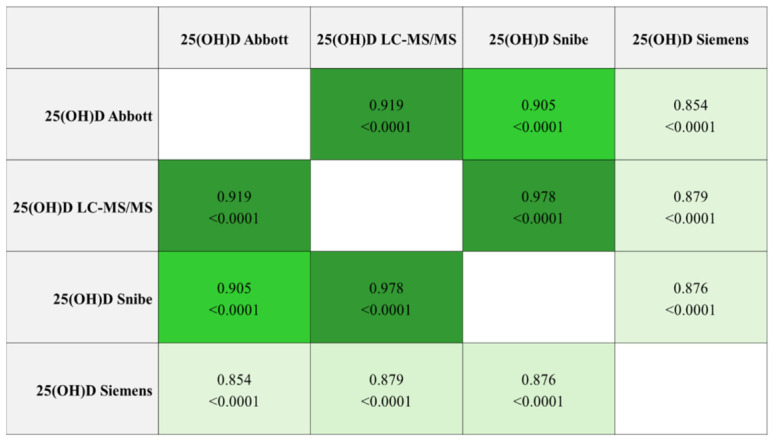
Heat-map of the overall sample (*N* = 425) between the 4 assays. The Spearman coefficient (r) for each correlation is reported. Colour legend: darker green indicates a higher correlation; lighter green indicates a lower correlation.

The heat-map between the 25(OH)D levels from each assay is shown in Figure 1 (*N* = 425) and provides a comparative analysis of their performance.

**Figure 2:**
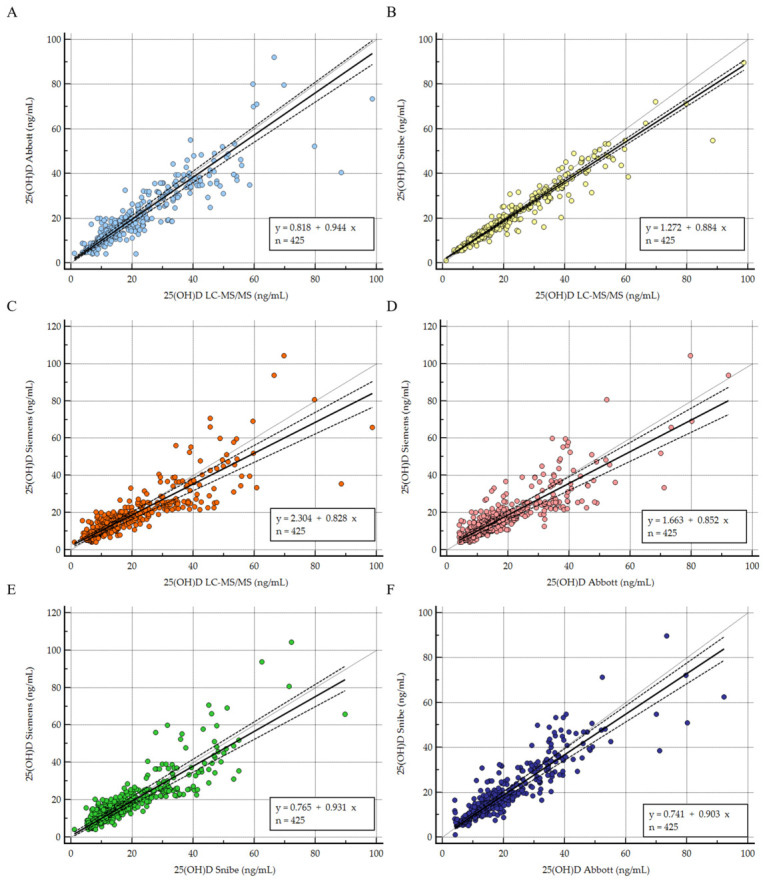
Passing–Bablok correlations among LC-MS/MS and three CLIA platforms for 25(OH)D measurement. (**A**) Passing–Bablok correlation between LC-MS/MS and Abbott CLIA. (**B**) Passing–Bablok correlation between LC-MS/MS and Snibe CLIA. (**C**) Passing–Bablok correlation between LC-MS/MS and Siemens CLIA. (**D**) Passing–Bablok correlation between Abbott CLIA and Siemens CLIA. (**E**) Passing–Bablok correlation between Snibe CLIA and Siemens CLIA. (**F**) Passing–Bablok correlation between Abbott CLIA and Snibe CLIA. [Solid line is the regression line; dashed lines represent the %95 CI].

Method agreement was evaluated using Passing–Bablok regression analysis. As shown in Figure 2A, the analysis between LC-MS/MS (x-axis) and Abbott assay (y-axis) demonstrated a regression line of y = 0.818 + 0.944x (95% CI for intercept: 0.225 to 1.313; 95% CI for slope: 0.899 to 0.996) and a strong correlation between the two methods, with a Spearman coefficient of r = 0.919 (*p* < 0.0001; 95% Confidence Interval, CI: 0.903 to 0.932). Similarly, Figure 2B illustrates the correlation between LC-MS/MS and Snibe, yielding a regression line of y = 1.272 + 0.884x (95% CI for intercept: 0.992 to 1.552; 95% CI for slope: 0.866 to 0.903) and an excellent Spearman coefficient of r = 0.978 (*p* < 0.0001; 95% CI: 0.974 to 0.982). Furthermore, the comparison between LC-MS/MS and the Siemens assay (Figure 2C) showed a regression line of y = 2.304 + 0.827x (95% CI for intercept: 1.294 to 3.063; 95% CI for slope: 0.764 to 0.885) and a robust relationship with a Spearman coefficient of r = 0.879 (*p* < 0.0001; 95% CI: 0.855 to 0.899).

Inter-assay comparisons were also performed, demonstrating a similar trend in all the chemiluminescent assays: the Siemens assay was compared against Abbott (Figure 2D), showing a regression line of y = 1.663 + 0.852x (95% CI for intercept: 0.612 to 2.545; 95% CI for slope: 0.783 to 0.919) and Spearman coefficients of r = 0.854 (*p* < 0.0001; 95% CI: 0.826 to 0.878), while against Snibe (Figure 2E) a regression line of y = 0.765 + 0.931x (95% CI for intercept: −0.007 to 1.741; 95% CI for slope: 0.872 to 1.000) and r = 0.876 (*p* < 0.0001; 95% CI: 0.851 to 0.896). Finally, the Snibe assay was compared against Abbott (Figure 2F), resulting in a regression line of y = 0.741 + 0.903x (95% CI for intercept: 0.181 to 1.288; 95% CI for slope: 0.853 to 0.956) and a Spearman coefficient of r = 0.905 (*p* < 0.0001; 95% CI: 0.886 to 0.920).

For all the platforms, no constant or proportional bias was observed.

**Figure 3:**
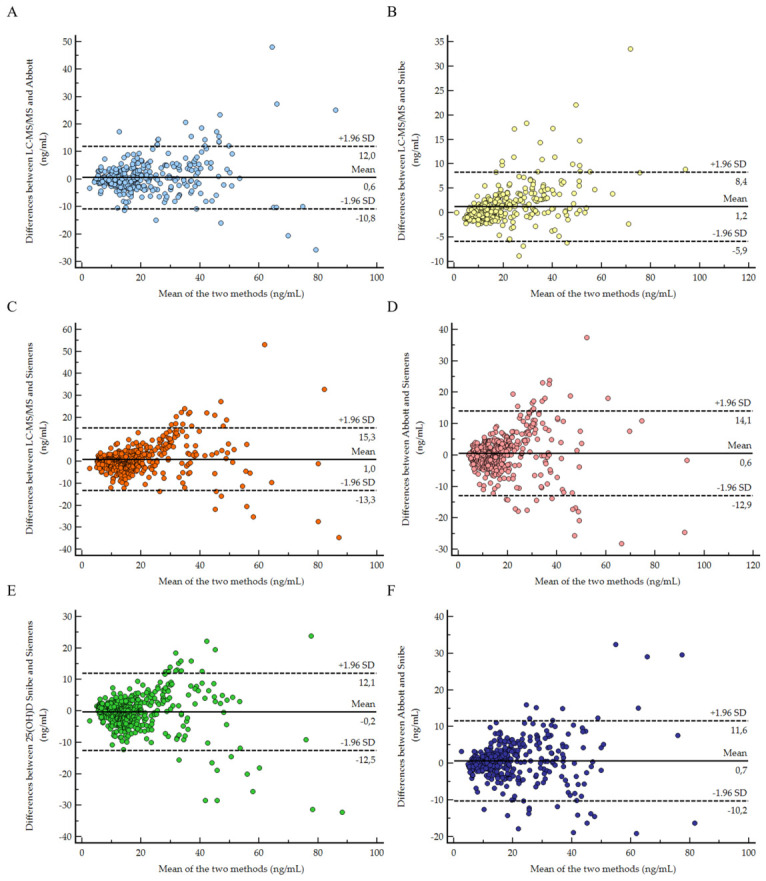
Bland–Altman analysis of agreement between LC-MS/MS and three CLIA platforms for 25(OH)D measurement, and among the CLIA methods. (**A**) Bland–Altman plot between LC-MS/MS and Abbott. (**B**) Bland–Altman plot between LC-MS/MS and Snibe. (**C**) Bland–Altman plot between LC-MS/MS and Siemens. (**D**) Bland–Altman plot between Abbott and Siemens. (**E**) Bland–Altman plot between Snibe and Siemens. (**F**) Bland–Altman plot between Abbott and Snibe. [Solid line represents the mean bias; dashed lines represent the %95 CI].

Additionally, method agreement was characterized via Bland–Altman analysis. As illustrated in Figure 3A, the mean bias between LC-MS/MS and the Abbott assay was 0.6 ng/mL, with 95% limits of agreement (LoA) ranging from −10.8 to 12.0 ng/mL. In contrast, the comparison between LC-MS/MS and Snibe in Figure 3B revealed a slightly higher mean bias of 1.2 ng/mL (95% LoA: −5.9 to 8.4 ng/mL). Similarly, the analysis for the Siemens assay against the reference LC-MS/MS method (Figure 3C) showed a mean bias of 1.0 ng/mL (95% LoA: −13.3 to 15.3 ng/mL).

Notably, the inter-assay comparisons demonstrated that all methods are highly comparable, with strong agreement and mean biases near zero. Specifically, the bias was 0.6 ng/mL for Abbott vs. Siemens in Figure 3D (95% LoA: −12.9 to 14.1 ng/mL) and 0.2 ng/mL for Snibe vs. Siemens in Figure 3E (95% LoA: −12.5 to 12.1 ng/mL). Finally, the agreement between Abbott and Snibe (Figure 3F) yielded a mean bias of 0.7 ng/mL (95% LoA: −10.2 to 11.6 ng/mL).

Across all comparisons, most data points remained within the 95% limits of agreement, indicating that these methods exhibit low disagreement with the LC–MS/MS gold standard over the tested concentration range, despite a slight increase in variability at higher concentrations and LoA relatively wide, but comparable.

### 3.3. Precision Study

Precision was evaluated by analyzing 3 replicates for 5 consecutive days (3 × 5 protocol). The precision profiles of the three analytical platforms (Abbott, Siemens, and Snibe) were assessed in Table 2 using Quality Controls by the manufacturer, focusing on intra-run, inter-run, and total precision expressed as the coefficient of variation (CV%).

All three platforms met the general requirements for analytical quality, with total precision CVs consistently remaining below the 5% threshold. The Abbott platform showed the most favorable precision profile at high concentrations, while Siemens demonstrated superior repeatability at lower levels. The Snibe system confirmed a balanced precision across the two evaluated levels.

### 3.4. Analytical Assay Performance

The analytical performance of all evaluated systems was found to comply with internationally accepted criteria for clinical acceptability (TEa < 21.5%). Imprecision was estimated from QC data, while bias was derived from the mean bias of 12 samples EQA results: Siemens mean bias −0.333%; Abbott mean bias −0.743%; Snibe mean bias −0.539. To ensure a conservative evaluation, the highest %CV observed for each system was used for TE calculation in the σ formula, according to a ‘worst-case scenario’ approach.

As shown in Table 3, the Siemens platform exhibited a maximum TE of 6.83% at low concentration levels, corresponding to a Sigma metric of 3.84. Comparable results were observed for the Abbott system, which showed a maximum TE of 6.23% and a Sigma value of 3.84. Among the platforms tested, the Snibe system demonstrated the best performance in the low concentration range, with higher TE (5.57%) and the highest Sigma metric (4.14). Notably, all calculated TE values were well below the predefined allowable total error threshold of 21.5%, indicating that each system meets the minimum analytical performance requirements.

### 3.5. Inter-Rater Agreement on the Vitamin D Status

The clinical agreement between the reference LC-MS/MS method and the three investigated immunoassays (Siemens, Snibe, and Abbott) for categorizing Vitamin D status is detailed in Table 4. This table summarizes the cross-classification of patient samples across the different clinical categories for each platform.

To account for the ordinal nature of the Vitamin D classes, Cohen’s weighted kappa analyses were performed to evaluate the strength of agreement. The reference method and all three immunoassays demonstrated a very good and similar agreement, 0.921 for Siemens (0.894 to 0.948), 0.928 for Snibe (0.903 to 0.953), and 0.926 for Abbott (0.903 to 0.952).

## 4. Discussion

In this study, we compared three automated CLIA platforms with the LC–MS/MS reference method for the measurement of serum 25(OH)D in a large cohort of postmenopausal and menopausal women, a population characterized by a high prevalence of vitamin D deficiency [22] and using a rapid and easy-to-implement method for 25(OH)D measurement is essential to support efficient clinical workflows and timely patient management [2,23,24].

All three CLIA platforms demonstrated excellent analytical performance, characterized by strong correlations with the gold-standard LC–MS/MS [25] and high precision. Notably, the Snibe platform showed the highest level of agreement, followed closely by Abbott and Siemens [26]. This stronger correlation may be attributable, at least in part, to its immunoassay design, including the different principles utilized for analyte detection. Beyond these strong associations, these immunoassays exhibited remarkable precision, with low intra- and inter-assay coefficients of variation (CV%) that fall well within the established clinical CLSI requirements (<5%) [15]. These findings support the robustness of the evaluated CLIA systems for routine laboratory use. Such performance characteristics are crucial to process large sample volumes with reduced turnaround times while maintaining analytical quality, especially when assays are used for longitudinal monitoring, where consistency over time directly impacts clinical interpretation and decisions.

Bland–Altman analysis confirmed that mean biases were generally small, but the limits of agreement were relatively wide in some comparisons and may be clinically relevant near diagnostic thresholds, particularly evident for Siemens versus LC–MS/MS. This variability was more pronounced at higher concentrations, a trend consistent with previous literature [27,28,29]. Rather than limiting their utility, these results reinforce the suitability of modernized CLIAs for routine screening; however, still the LC-MS/MS remains the gold standard. The ongoing standardization of Vitamin D assays has significantly narrowed the gap between automated platforms and reference methods, facilitating a more unified approach to patient care.

While earlier generations of assays faced challenges with cross-reactivity and calibration drift, the ongoing efforts of the Vitamin D Standardization Protocol (VDSP) have significantly harmonized results across automated platforms, reinforcing their suitability for routine screening [30,31]. These harmonization initiatives have contributed to improved comparability of results at both national and international levels, supporting the development of standardized clinical guidelines.

Furthermore, given the high prevalence of deficiency in the study cohort (88%: 374 patients below the normal status range 30 ng/mL), even modest analytical discrepancies may lead to clinically meaningful misclassification, especially near diagnostic thresholds.

The Sigma metrics obtained (Abbott 3.84, Siemens 3.84, Snibe 4.14) indicate an overall acceptable level of analytical performance. According to widely accepted quality benchmarks, Sigma values between 3 and 4 are indicative of moderate analytical performance, requiring appropriate quality control strategies to ensure reliability [32]. However, these results should be interpreted in the context of the specific study population. In this context, the observed Sigma values can be considered satisfactory, as they demonstrate the ability of the evaluated systems to maintain acceptable analytical performance even in low concentration ranges. Nevertheless, residual differences between methods remain and may contribute to variability in clinical classification, particularly near decision thresholds; in cases of discrepancies or in some specific populations (i.e., children), the use of LC-MS/MS is always suggested.

This analytical reliability is particularly critical for menopausal and post-menopausal women, where vitamin D deficiency is widespread and closely linked to osteoporosis and sarcopenia. In these patients, vitamin D is essential not only for skeletal mineralization but also for maintaining muscle strength and preventing falls. The efficiency of standardized CLIA platforms allows for the precise longitudinal monitoring required to reach and maintain optimal 25(OH)D targets (typically >30 ng/mL or 75 nmol/L in high-risk groups). Although each immunoassay misclassified some patient values into the various categories, the high concordance and the ‘very good agreement’ demonstrated by the weighted kappa coefficients confirm that they are highly reliable for routine clinical stratification of Vitamin D status when compared to the LC-MS/MS gold standard.

By facilitating consistent screening, these assays empower clinicians to implement timely supplementation and anti-osteoporotic therapies, ultimately improving musculoskeletal health and reducing fracture risk during the menopausal transition [9,33]. In addition, early identification and correction of vitamin D deficiency may contribute to broader health benefits, including potential improvements in immune function and chronic fatigue. Overall, all evaluated immunoassays demonstrated satisfactory analytical performance, showing high levels of precision and agreement. While each platform exhibited specific strengths, their suitability for routine clinical use ultimately depends on the requirements and operational characteristics of the individual laboratory setting.

However, this study has several limitations. First of all, this is a monocentric evaluation, and the period covered represents just two months (February and March), which may not fully capture the seasonal variability of vitamin D status. However, since the primary aim was the analytical comparison of different methods rather than the assessment of vitamin D epidemiology, this aspect is unlikely to have significantly influenced the overall comparison between assays. Moreover, since the study was based on residual samples, information on vitamin D supplementation, osteoporosis treatment, renal disease, liver disease, malabsorption, obesity, and relevant medications is missing. Nevertheless, the large sample size and the comparison with the LC–MS/MS reference method provide a robust framework for assessing the analytical performance of the evaluated platforms.

## 5. Conclusions

Overall, the three CLIA platforms showed strong correlation and acceptable agreement with LC–MS/MS, but with some discrepancies and variability among them. These findings highlight the need for continued harmonization efforts and reinforce the importance of method-specific interpretation of 25(OH)D results in postmenopausal women [34] to ensure accurate clinical decision-making. Future research should focus on further refining assay standardization and evaluating the clinical impact of assay variability in diverse patients, thereby enhancing the reliability of vitamin D assessment in everyday practice.

## Figures and Tables

**Table 1:** Stratification of the population based on their 25(OH)D levels from Siemens assay (routine method). Values of the related calcium and parathormone [mean value (minimum–maximum)].

25(OH)D Status [20,21]	Range (ng/mL)	*N*	25(OH)D (ng/mL)	Calcium (mmol/L)	Parathormone (ng/L)
Severe deficiency	<10	83	6.98 (4.20–9.98)	2.40 (2.02–2.74)	115.0 (52.2–304.0)
Moderate deficiency	10–20	211	15.50 (11.00–19.9)	2.43 (1.85–2.72)	90.0 (41.6–473.0)
Insufficiency	20–30	80	25.40 (21.10–29.90)	2.43 (2.05–2.89)	71.5 (25.2–249.0)
Normal status	30–100	50	41.50 (31.00–93.81)	2.43 (1.85–2.84)	38.2 (36.0–87.9)
Toxicity	>100	1	108.00	2.5	47.7

**Table 2:** Precision study evaluated through the 3 × 5 protocol, using quality controls, of the 3 immunoassays. Mean and Standard Deviation (SD) are expressed in ng/mL. In grey, the observed Coefficients of Variation (%CV) are highlighted.

		Control 1			Control 2			Control 3		
		Mean	SD	%CV	Mean	SD	%CV	Mean	SD	%CV
**Abbott**	Intra-run	7.76	0.25	3.20	16.50	0.35	2.10	36.20	1.21	3.35
	Inter-run	7.80	0.23	3.01	16.72	0.54	3.26	35.30	1.31	3.70
	Total	7.78	0.26	3.33	16.83	0.49	2.93	35.33	1.41	3.98
**Siemens**	Intra-run	39.37	0.13	0.32	81.29	1.33	1.63			
	Inter-run	38.43	0.71	1.84	82.06	2.80	3.41			
	Total	38.47	1.47	3.82	81.03	2.81	3.47			
**Snibe**	Intra-run	21.35	0.28	1.30	51.28	0.95	1.85			
	Inter-run	21.04	0.59	2.82	53.23	1.64	3.07			
	Total	21.15	0.53	2.52	52.16	2.01	3.85			

**Table 3:** Evaluation of the Sigma metric for the three chemiluminescence assays (CLIA). The Sigma was evaluated by the formula σ = (TEa-|Bias%|)/(%CV), assuming the highest %CV observed for each assay. [QC: Quality Controls, CV: Coefficient of Variation; TE: Total Error].

CLIA Assay	QC Level	CV (%)	TE (%)	Sigma
Abbott	L1	3.33	4.18	3.84
	L2	2.93	3.53	
	L3	3.98	6.23	
Siemens	L1	3.82	6.83	3.84
	L2	3.47	5.45	
Snibe	L1	2.52	3.37	4.14
	L2	3.85	5.57	

**Table 4:** Comparison and Categorical Agreement of the 25(OH)D status of the population considered between the different platforms and the evaluation of Cohen’s weighted kappa (LC-MS/MS vs. the three immunoassays). [Weighted Kappa (95% Confidence Interval)].

25(OH)D Status	Severe Deficiency	Moderate Deficiency	Insufficiency	Normal Status	Toxicity	Cohen’s Kappa (95% CI)
LC-MS/MS	99	183	85	57	1	--
Siemens	83	211	80	50	1	0.921 (0.894 to 0.948)
Snibe	91	208	69	57	0	0.928 (0.903 to 0.953)
Abbott	85	206	81	53	0	0.926 (0.903 to 0.952)

## Data Availability

Data will be shared upon reasonable request by the corresponding author.

## Supplementary Materials

The following supporting information can be downloaded at: https://www.mdpi.com/article/10.3390/diseases14070245/s1. Figure S1: Schematic diagram of specific recognition of immune complexes by 25(OH)D non-competitive method.
