# Supplementary material for: Circulating 25-Hydroxy-Vitamin D Levels in Menopausal and Postmenopausal Women in Italy: A Comparison of Four Analytical Methods
Source: Diseases. 2026 Jul 6;14(7):245. doi: 10.3390/diseases14070245 (PMC13408729; doi:10.3390/diseases14070245)
Supplement: Supplementary file 1 [file diseases-14-00245-s001.zip › diseases-4376772-supplementary.pdf]

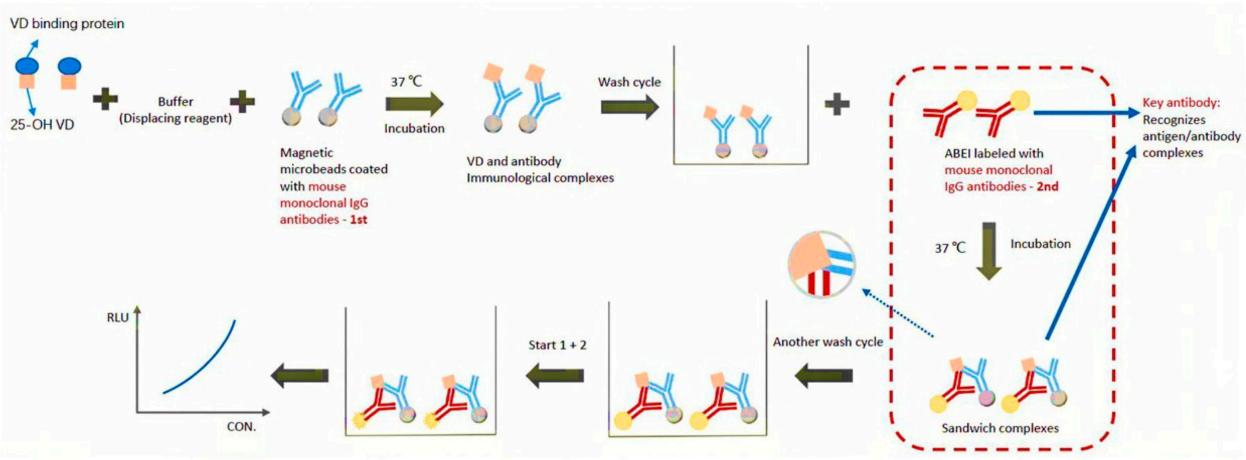

Figure S1. Schematic diagram of specific recognition of immune complexes by 25(OH)D non-competitive method.
